# Effect of packaging and encapsulation on the oxidative and sensory stability of omega‐3 supplements

**DOI:** 10.1002/fsn3.3182

**Published:** 2022-12-21

**Authors:** Hande Yenipazar, Neşe Şahin‐Yeşilçubuk

**Affiliations:** ^1^ Department of Food Engineering, Faculty of Chemical‐Metallurgical Engineering Istanbul Technical University Istanbul Turkey

**Keywords:** consumer behavior, omega‐3 fatty acids, oxidative stability, packaging, sensory analysis, storage study

## Abstract

Omega‐3 fatty acid consumption is getting more common due to its positive impacts on human health. Since consumers cannot get their omega‐3 needs from natural sources, omega‐3‐rich products play an essential part in the diet. However, they are highly susceptible to oxidation; thus, storage conditions affect their quality. Product form is also another critical factor for stability. In this study, fatty acid composition, oxidative stability, and sensory properties of different omega‐3 products having varied packaging types were investigated. Moreover, the effect of consumer behavior regarding the recommended usage was assessed during storage. Syrup forms (maximum values at the end of the storage: PV = 44.6 meq/kg oil for S32, *p*‐AV = 16.87 for S22, and TOTOX = 96.94 for S11) are more susceptible to oxidation than capsule (maximum values at the end of the storage: PV = 7.62 meq/kg oil for C31, *p*‐AV = 19.58 for C12, and TOTOX = 30.44 for C12) and chewable forms (maximum values at the end of the storage: PV = 26.14 meq/kg oil for G12, p‐AV = 13.47 for G12, and TOTOX = 65.76 for G12). In addition, capsules complied more with limit values during storage and were better protected according to the sensory scores. The aroma and taste of the omega‐3 products generally changed in a negative manner during storage. Capsulated samples were better protected according to the sensory evaluation scores at the end of the storage period. Fish oil samples belonging to the same company but provided from different stores showed significant differences, which is an indicator of nonstandard raw material, ingredient, or processing.

## Graphical abstract

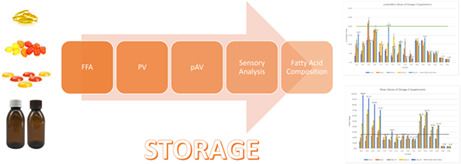

## 1 INTRODUCTION

The main source of long‐chain (LC) omega‐3 fatty acids are oily fish, fish oils, and some algae extracts, and they have numerous health benefits such as reducing the risk of cancer, heart disease, inflammation, arthritis, diabetes, and high cholesterol (Hong et al., 2011; Mazza et al., 2007; Saini & Keum, 2018). Eicosapentaenoic acid (EPA) is a 20‐carbon‐long omega‐3 fatty acid, and the most important of EPA's main functions is its use to create signaling molecules called eicosanoids which can reduce inflammation (Siriwardhana et al., 2012). EPA has been shown to be particularly effective against depression (Sublette et al., 2011). Docosahexaenoic acid (DHA) is another important omega‐3 fatty acid containing 22 carbons and its main role is to serve as a structural component in cell membranes, particularly nerve cells in the brain and eyes. It makes up about 40% of the polyunsaturated fats in the brain (Singh, 2005).

Daily consumption of fish oil is recommended in different amounts by different scientific authorities. While the European Food Safety Authority (EFSA) Panel on Dietetic Products, Nutrition and Allergies (NDA) (2012) recommends consuming 250–500 mg/day of EPA and DHA for adults with cardiovascular risk, scientific authorities recommend the consumption of fish twice a week to benefit from the nutritional effects of omega‐3 fatty acids (Scientific Advisory Committee on Nutrition, 2004). However, omega‐3 fatty acids are not adequately consumed by the majority of the population (Lane & Derbyshire, 2018). Generally, consumers get their omega‐3 needs from daily fish oil and omega‐3 supplements, not from natural sources of oily fish.

In addition to the unique EPA and DHA content of fish oils, these omega‐3 fatty acids are the fatty acids that are most susceptible to oxidation. From the moment the bottles of the fish oil supplements are opened, they are getting exposed to a process called “oxidation” which starts with the contact of oxygen in the air and results in rancidity and deterioration problems (De Boer et al., 2018). At the end of this process, taste and odor deterioration occur in fish oil. Due to the oxidation problem, the nutritional value of oils decreases, compounds such as 4‐hydroxy‐2‐alkenal and 4‐hydroxy‐2‐hexanal are formed which negatively affect health, unwanted odor and taste form, and the shelf life reduces, which are the problems associated with oxidation (Bartee et al., 2007). With oxidation, losses occur in essential fatty acids; therefore, it is important that oils containing omega‐3 fatty acids have acceptable sensory quality and oxidative stability.

In order to prevent spoilage, oils should be stored at temperatures as low as possible and protected from light. With the use of antioxidants, which is the method used to prevent oxidative reactions, it will be possible to capture the peroxide radicals formed at the beginning of the oxidative reactions before the chain reactions start (Miyashita et al., 2018).

It is recommended to encapsulate these oils in various polymer matrices (Let et al., 2004; Nielsen et al., 2004). Sunflower phospholipids, whey protein concentrate, maltodextrin, β‐lactoglobulin, fibrils, and chitosan were examples of the used polymer matrix (Chang et al., 2020; Chen et al., 2020). In addition to masking the taste with encapsulation technology, protective properties are also provided. It was proved that using encapsulation systems (such as gels, emulsions, colloidal forms, and powders) were effective on the physicochemical and oxidative stability of omega‐3‐rich oils. Spray, freeze, and fluidized bed‐drying operations could be applied to convert the colloidal materials into powdered forms (Venugopalan et al., 2021). Moreover, the selection of an appropriate encapsulation technology enables to overcome low bioavailability and low water solubility of the omega‐3 fatty acids (Du et al., 2022). By the encapsulation of oils containing omega‐3 fatty acids, controlled release of omega‐3 fatty acids, which are lipophilic functional ingredients, during digestion can also be achieved (Chang & Nickerson, 2018).

According to our research, up to date, there is no storage study including consumer behavior regarding the recommended usage after the bottles are opened, and there is no study examining the chemical and sensory changes that occur during storage period. The accuracy of the label information in terms of omega‐3 fatty acids of the products purchased from the market and chemical analysis at the time the products were opened have only been investigated (Albert et al., 2015; Damerau et al., 2020; Heller et al., 2019; Özyurt et al., 2013).

In this project, it is aimed to compare the effects of different forms (capsule, chewable tablet, and syrup) and packaging types of omega‐3 products on fatty acid composition, oxidative stability, and sensory properties when stored at room temperature and in dark conditions. The most preferred capsules, syrups, and chewable tablets in the Turkish market were purchased and the products were stored in their own packaging at room temperature and out of light. In order to mimic consumer usage, syrup samples were reduced by 5 ml/day, which is the recommended daily dose, whereas capsules and chewable tablets have also been reduced considering recommended consumption. It aimed to correlate the oxidation parameters of omega‐3 products with the sensory properties by performing comparative analysis once a week, including the initial status of the oils before storage.

## 2 MATERIALS AND METHODS

### 2.1 Reagents and standard

Analytical‐grade chemicals were used for all analyses. For the peroxide value (PV) analysis, potassium iodide (Sigma Aldrich, Steinheim, Germany), sodium thiosulfate (Merck, Darmstadt, Germany), potato starch (Sigma Aldrich, Steinheim, Germany), SDS (Merck, Darmstadt, Germany), and isooctane (Isolab, Wertheim, Germany) were employed. The *p*‐anisidine value (*p*‐AV) reagents included *p*‐anisidine (Sigma Aldrich, Steinheim, Germany) and glacial acetic acid (Isolab, Wertheim, Germany). Isopropyl alcohol, toluene, and phenolphthalein were employed in free fatty acids (FFA) analysis. Chloroform (Isolab, Wertheim, Germany) and methanol (Riedel‐de Haën, Seelze, Germany) were used in the oil extraction procedure from gel samples. A standard consisting of 37 FAMEs including EPA and DHA was purchased from Supelco (Sigma Aldrich, Poole, UK).

### 2.2 Sample materials

In order to explore the effect of product type on the quality of omega‐3 products, three different types of omega‐3 products, including syrup, capsule, and chewable forms, were chosen. They were selected according to the highest sales figures in the Turkish market and purchased from local pharmacies. For each category, products of three different brands (denoted by different numbers in decimal place) were investigated weekly during their consumption period. Moreover, to provide independent repetitions of samples, two products having different lot numbers of the same brand (denoted by different numbers in the ones place) were analyzed. Samples were named in terms of their form and brand. For instance, for sample S12, “S” stands for syrup, “1” was for the brand, and “2” was for the different lot number. Capsule forms were denoted as C, and chewable forms were denoted as G. Properties of omega‐3 products and their analysis periods are given in Table 1. Consumer behavior was mimicked during the storage period. Suggested consumption amounts were discarded daily from each sample. Since only 6.8% of omega‐3 supplement users store the products in refrigerated conditions (Mengelberg et al., 2018), the samples were stored at room temperature during storage period. Comparative oxidation analysis and sensory analysis were performed once a week, including the properties at the time of first opening. The fatty acid composition of fish oils was analyzed at the first opening and at the end of storage to investigate any changes.

**TABLE 1:** Details of omega‐3 products and analysis period

Sample	Product Type	Package Type	Ingredients	Flavor	Amount	Storage period	Analysis
S1	Syrup	Amber glass	Fish oil (in triglyceride form), natural flavor, antioxidant tocopherol‐rich extract (vitamin E)	Orange	150 ml	30 days	5 weeks
S2	Syrup	Amber glass	Fish oil, natural orange flavor, antioxidant: α‐tocopherol‐rich extract	Orange	150 ml	30 days	5 weeks
S3	Syrup	Amber glass	Cod liver oil (97.1%), natural lemon flavor (2.7%), DL‐α‐tocopherol acetate, tocopherol rich extract, retinil palmitate, cholecalciferol	Lemon	250 ml	50 days	8 weeks
C1	Capsule	Amber glass	Fish oil, fish gelatin, glycerol, distilled water, lemon oil, antioxidant tocopherol‐rich extract (vitamin E)	Lemon	50 capsules	30 days	5 weeks
C2	Capsule	Amber glass	Fish oil concentrate, fish oil antioxidant tocopherol‐rich extract, bovine gelatin, glycerin	‐	50 capsules	30 days	5 weeks
C3	Capsule	Plastic	Fish oil (77.6%) and bovine gelatin as capsule material, glycerol, antioxidant tocopherol rich extract	‐	50 capsules	30 days	5 weeks
G1	Chewable Capsule	Individual plastic +Aluminum	Fish gelatin, glycerol, tapioca starch, water, D‐α‐tocopherol, tutti‐frutti flavor, orange flavor, sunflower lecithin, cholecalciferol, sucralose	Tutti‐frutti and Orange	30 chewable forms	30 days	5 weeks
G2	Chewable Gum	Aluminum pouch	Glucose syrup, sugar, apple juice (7%), bovine gelatin, water, algae oil, citric acid, pectin, natural flavors, anthocyanin, paprika extract, lutein, tocopherol‐rich extract, sucralose	Different flavors	60 chewable forms	30 days	5 weeks
G3	Chewable soft gel	Individual blaster	Fish oil concentrate, xylitol, sorbitol, water, bovine gelatin, sodium citrate, malic acid, natural orange and lemon flavors, cholecalciferol, carotene	Orange and lemon	30 chewable forms	30 days	5 weeks

### 2.3 Sample preparation

Syrup samples were analyzed without any sample preparation step, and oil from capsules and G1 chewable samples was collected with a needle and syringe immediately before analysis. Lipid extraction took place before the analysis of chewable samples, G2. Firstly, six gel forms were dissolved in distilled water (1:10, w/v) using IKA brand KS4000i orbital shaker (Germany) at 300 rpm for 1 h. This step enabled to loosen gelatin structure and ease of lipid extraction from the gel matrix. The liquid layer was discarded and solid particles were collected for extraction procedure. Afterward, Folch method was applied and samples were dissolved into 20‐fold chloroform: methanol (2:1 v/v) solution using IKA brand KS4000i orbital shaker (Germany) at 300 rpm for 2 h. Supernatants were collected, and the extraction procedure was repeated with the residues in order to prevent oil loss. Distilled water was added to supernatants and the mixture was centrifuged at 4000 *g* at 4°C for 5 min (Ren et al., 2017). Lower layer was used for further analysis after the evaporation of the solvent under vacuum using rotary evaporator (IKA RV10, Germany).

G3 samples were manufactured using ConCordix technology; thus, a different procedure was applied for lipid extraction from these samples. Twenty grams of sample was placed onto a drying paper sheet to remove the residuals of oil and cut into smaller pieces. The cut material was placed into a 250‐ml glass container and closed. 20 ml chloroform and 40 ml methanol were mixed and added to the sample. The mixture was homogenized for 120 s with a rotor stator homogenizer (Ultra‐Turrax, IKA T18, Germany) at 6500 rpm. 20 ml chloroform was added and ultra‐Turrax mixing continued for 60 s. 32.6 ml distilled water was added and the mixture was swirled two or three times. The bottom chloroform layer was collected in a 50‐ml tube and centrifuged for 3 min at 5250 *g* at room temperature. The residues were discarded. Chloroform was evaporated under vacuum using rotary evaporator (IKA RV10, Germany).

### 2.4 Fatty acid composition

To prepare the fatty acid methyl esters (FAME) for gas chromatography, 0.1 g of samples was weighed and 3 ml of 6% HCl in methanol solution was added to samples. The samples were vortexed for a minute and later incubated in the oven at 75°C for 2 h. After incubation, samples were chilled on ice for 5–10 min. 2 ml hexane and 1 ml of 0.1 M KCl were added. The samples were vortexed for a minute and later centrifuged at 1000 rpm for 3 min. The upper hexane layer was collected and passed through an anhydrous sodium sulfate column to remove water. Steps after adding hexane were repeated and collected hexane layers were combined (Yüksel & Şahin‐Yeşilçubuk, 2012).

Fatty acid compositions of omega‐3 products were analyzed by using Agilent Technologies 6890 N gas–liquid chromatography (GLC) equipped with a flame‐ionization detector and Supelco SP‐2380 capillary column (60 m × 0.25 mm ID × 0.20 μm film thickness) (Supelco, CA). The injector and detector temperatures were held at 240 and 250°C, respectively. The oven temperature was initially held at 150°C for 3 min, and was then programmed to 225°C for 15 min at a rate of 10°C/min and the total flow rate was 25.8 ml/min. A 1 μl sample was injected into the GLC. Average values from the results of duplicate analyses were reported (Yüksel & Şahin‐Yeşilçubuk, 2012).

A standard consisting of Supelco 37 FAMEs including EPA and DHA was used as an external standard.

### 2.5 Free fatty acid content

Free fatty acids of omega‐3 samples were determined by a titrimetric method. AOCS Official Method Cd 3d‐63 was applied for FFA determination (AOCS Official Method Cd 3d‐63, 2004).

### 2.6 Oxidative stability

In order to compare the effects of different forms (capsule, chewable, and syrup) and packaging types of omega‐3 products on oxidative stability; PV and *p*‐AV analysis tests were conducted. AOCS procedures were applied for oxidative stability analysis. PV was determined according to AOCS Official Method Cd 8b‐90 (AOCS Official Method Cd 8b‐90, 2004) by visual titration of iodine. For *p*‐AV determination, AOCS Official Method Cd 18–90 was employed (AOCS Official Method Cd 18‐90, 2004). The total oxidation (TOTOX) value of the samples was calculated using Equation 1 below.

(1) $$\text{TOTOX}=\left(2\times \mathrm{PV}\right)+\mathrm{PAV}$$

### 2.7 Sensory analysis

Sensory analysis of samples was carried out by eight trained panelists using quantitative descriptive analysis (QDA) method. Before starting the panel, a training session was performed to practice terminology development, the use of references, and the scale for each type of sample group, individually. A structured intensity scale with a range from a minimum of 0 to a maximum of 9 was used. Samples (2 ml of syrup, one capsule, or one gel) were presented by coding with three‐digit numbers and served at room temperature together with water and crisp bread. All evaluations were made in duplicate. The sensory characteristics and the references used in the study are shown in Table 2.

**TABLE 2:** Sensory characteristics and the references used in sensory analysis

Descriptor	Definition	Omega‐3 form	Reference	Reference score
Citrus odor	Related to odor of citrus fruit	Syrup	Citrus fruit	9
Fruity odor	Related to odor of fruits	Chewable	Commercial jelly gum	7
Fish odor	Related to odor of fish	All	Fish oil	9
Rancid odor	Related to odor of oxidized oil	Syrup	Oxidized fish oil	
Foreign odor	Related to an undesirable/unexpected odor	All	NA	
Consistency	Related to viscosity of liquid	Syrup	Sunflower oil	4
Chewiness	Related to the chewy nature of the sample	Chewable	Commercial jelly gum	7
Acidity	Related to the taste of organic acids	Syrup	Lemon oil added Sunflower oil	4
Sweetness	Related to the sweet taste	All		
Rancid taste	Related to taste of oxidized oil	Syrup	Heat‐exposed fish oil	6
Citrus aroma	Related to aroma of citrus fruit	Syrup	Citrus fruit	9
Fruity aroma	Related to aroma of fruits	Chewable	Commercial jelly gum	7
Fish aroma	Related to aroma of fish	All	Fish oil	9
After taste	Related to taste and aroma after swallowing	Syrup, Chewable	NA	

### 2.8 Statistical analysis

The results were given as means ± standard deviation of three experimental repetitions for syrup samples and two experimental repetitions for capsule and gel forms. One factor analysis of variance (ANOVA) test was used for statistical analysis of the results, at the 95% level (*p* < .05) of significance. Also, Tukey test was applied to determine significant differences. In order to perform statistical analysis tests, MINITAB Statistical Software (version 16.1.0) (Minitab Ltd.) was used.

## 3 RESULTS AND DISCUSSION

### 3.1 Fatty acid composition

EPA and DHA contents (%) of commercial omega‐3 products used in this study varied between 18.4% in sample G2 and 69.1% in sample C2. There were no differences observed during storage period.

### 3.2 Free fatty acid content

**FIGURE 1:**
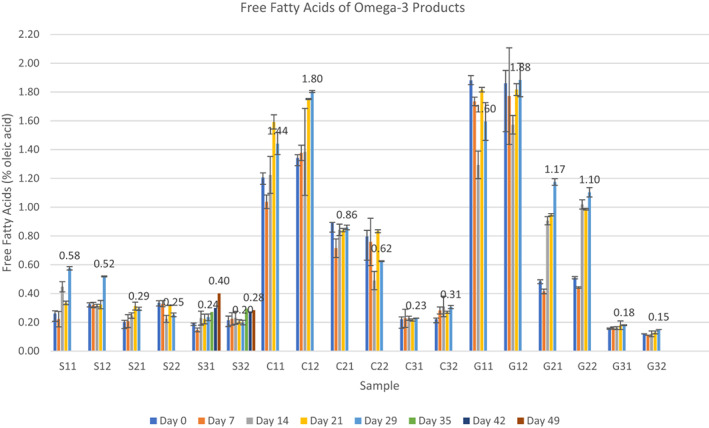
Free fatty acids of analyzed omega‐3 products in different forms.

FFA values indicating the hydrolytic rancidity of the omega‐3 products during storage period are reported in Figure 1. According to FFA results, the lowest FFA was detected in chewable G32 (0.11%) and the highest value in chewable capsule G12 (1.88%) on 29th day of storage.

The low levels of % FFA in fish oil samples investigated indicate that they were of good quality. As can be seen in Figure 1, the initial FFA values of the fish oil syrup samples were found to be lower than the other capsule and chewable forms. However, C3 coded capsule samples showed similar results with the syrup forms. In addition, the initial FFA values of the C1 and G1 coded samples were found to be relatively high compared to other omega‐3 products. It can be interpreted that different processing techniques can also affect the initial content of FFA. During long‐term storage period, increase in FFA content was especially high in the last week of storage in the S1 coded syrup samples, while other syrup samples were more stable.

In the C1 coded samples, it was seen that the FFA values increased as the storage time increased. FFA values in C2 and C3 coded samples were observed to be stable during storage period. In the examined chewable samples, it was seen that the FFA values of the G1 and G3 coded samples were stable during the storage period, and the FFA value of the G2 coded sample was in an increasing trend.

Some organizations give limits for acid value or FFA (oleic acid, %), while GOED provides guidance information for acid value limits for marine or fish oils (De Boer et al., 2018). FFAs are not only important from the point of view of oxidation products but also have been reported to have a direct sensory impact (Ashton, 2002).

### 3.3 Oxidative stability

**FIGURE 2:**
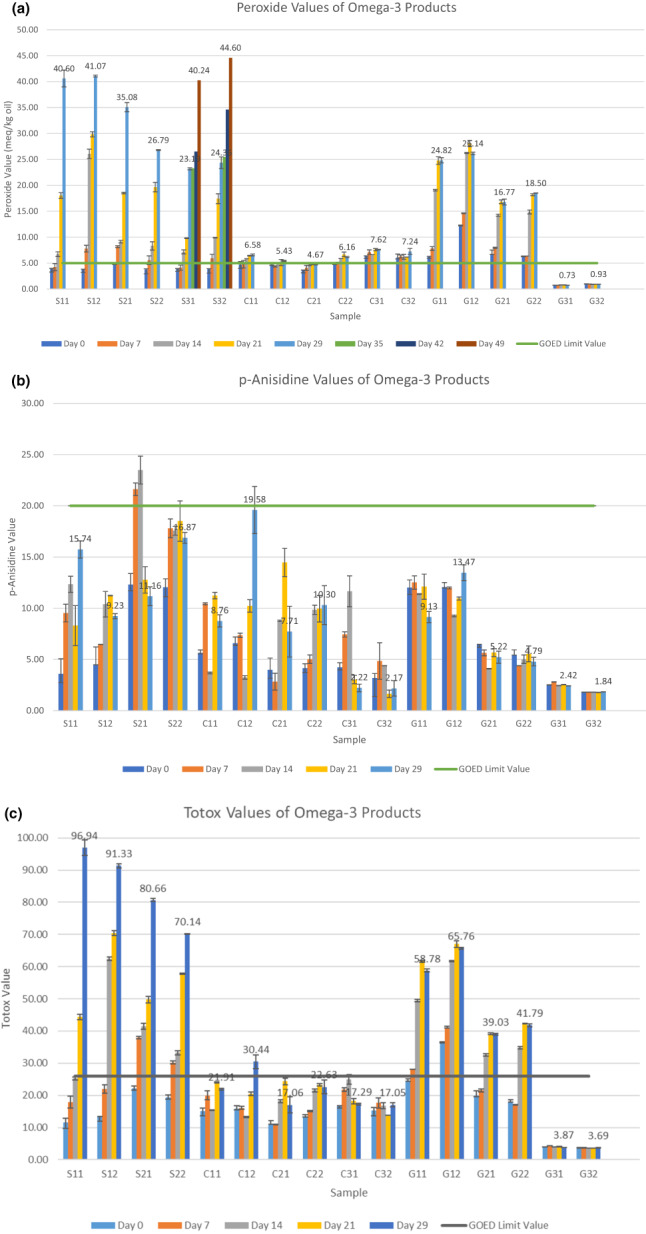
The alteration of peroxide (a), *p*‐anisidine (b), and TOTOX (c) values of omega‐3 products during storage.

The PV, *p*‐AV, and TOTOX values were measured for the samples during storage at room temperature (~25°C). The results are given in Figure 2. The PV indicates the formation of primary oxidation products, and the *p*‐AV method is used for the assessment of the secondary oxidation products of oils.

According to PV results (Figure 2a), the lowest PV was detected in chewable Gel 31 (0.73 meq/kg oil) at initial stage and the highest value in Syrup 32 (44.60 meq/kg oil) on 49th day of storage. Based on the peroxide values of the fish oil samples, it was observed that the peroxide values in the syrup forms had an increasing trend during the storage period, while a stable trend was observed in the capsule samples. In the chewable forms, it was observed that the peroxide values in the G1 and G2 coded samples had an increasing trend during the storage period, while a stable trend was observed in the G3 coded sample.

GOED provides limit value for peroxide value as 5 meq/kg oil for marine or fish oils (De Boer et al., 2018). Gracey and Collins (1999) reported that PV below 5 meq/kg oil indicates that fat is not oxidized, while PV between 5 and 10 meq/kg indicates rancidity. According to the limit of GOED, initial PV values of the three syrup samples were lower than 5 meq/kg oil. PV values of the capsule forms did not exceed the limit values; however, sample C3 had slightly higher PV values (*p* > .05). In terms of chewable samples, except G31 and G32 coded samples, PV values were slightly higher than the limit value during storage. The limit value was exceeded for samples C31, C32, G11, G12, G21, and G22 at the beginning of storage period (day 0) (*p* > .05; for G12: *p* < .05), whereas for S12, S21, S22, and S32 on the 14th day (*p* > .05), for samples S11, S31, C11, C12, and C22 at the 21st day (*p* > .05). During storage period, PV values of three capsule samples and G3 coded chewable gel sample were relatively stable compared with other fish oil samples. Capsules were more protected from environmental conditions and G3 coded chewable gel sample had more protection due to individual aluminum packaging. Syrup forms of fish oils had high PV values due to the exposure to pro‐oxidants such as atmospheric oxygen. Therefore, consumption of fish oil capsules and chewable in the gel form with individual blister packing might be safer than fish oil syrups with respect to oxidative stability.

As can be seen in Figure 2b, significant variance in *p*‐AV results between various types of oils has been observed. GOED recommends the analysis of *p‐*AV for fish oils, without ingredients other than antioxidants. Furthermore, *p‐*AV test is not appropriate for measuring secondary oxidation in fish oils with strong color such as carotenoids, astaxanthin, or oils including flavorings, which interfere with the *p*‐AV assay and further yield invalid results (Ismail et al., 2016). Some researchers noted that salmon oils containing high astaxanthin content had high levels of *p‐*AV due to interferences. Moreover, cod liver or fish oils including fruit‐derived flavors might pose similar interference problems. While performing anisidine value analysis, aldehyde derivative components, which are secondary oxidation products, are measured by spectrophotometric methods. Since flavors added to fish oils may also contain aldehyde‐derived components (such as citral in lemon flavor), they can react similarly to aldehydes formed by lipid oxidation and higher *p*‐anisidine value results can be obtained from nonflavored oils (Bannenberg et al., 2017; Damerau et al., 2020; Ismail et al., 2016; Nichols et al., 2016). In our study, all the samples except C2 and C3 samples had fruit flavors. It can be concluded that *p*‐AV measurement on oils with added lemon, orange, or tutti‐frutti flavor gave unreliable results. Especially, we could not get consistent results with the S3 coded fish oil syrup sample since it had high contents of natural lemon flavor, which was also stated in sensory analysis. This sample had a sharp lemon flavor and contained more intense citrus flavor than other syrups. As a consequence, the results are missing in Figure 2b due to interference problems during analysis. The limit value was exceeded for sample S21 on the 7th day.

The TOTOX value results are given in Figure 2c. TOTOX value is used to determine the total oxidative stability of the omega‐3 products by including primary and secondary oxidation measurements. Since we could not get results with S3 syrup forms due to high amount of lemon flavor, TOTOX calculation could not be employed for this sample. Although TOTOX value is widely employed to express oxidative quality of omega‐3 oils, it is not valid for oils containing interfering ingredients (flavors or colors) due to the unreliable results obtained by *p*‐AV (Ismail et al., 2016).

In our study, since anisidine values were found to be low in fish oil capsules (C2 and C3 samples) and chewable gel (G3 sample), TOTOX values were also found to be lower than GOED's limit value of 26 during the storage period. While the TOTOX values of fish oil syrups tended to increase depending on storage time with the exposure to oxygen, no significant change was observed in the individually packaged G3 coded chewable gel sample during storage period. The limit value was exceeded for samples G12 at the beginning of storage period (day 0), for samples S21, S22, and G11 on the 7th day, whereas for S12, G21, and G22 on the 14th day, for S11 on the 21st day, and C12 on the 29th day.

### 3.4 Sensory analysis

#### 3.4.1 Fish oil syrup

For fish oil syrup samples, more characteristics were evaluated compared to gel and capsule samples since textural attributes are more critical and needed to be covered in these samples. The sensory evaluation of fish oil syrup samples during the storage period is given in Table 3.

**TABLE 3:** sensory evaluation of fish oil syrups

TERMS	SAMPLE	DAYS							
		0	7	14	21	29	35	42	49
CITRUS ODOR	S11	4.40 ± 0.89 ^ABa^	4.40 ± 0.55 ^Aa^	4.20 ± 0.45 ^Aa^	4.20 ± 0.84 ^Aa^	4.20 ± 0.45 ^Aa^	NA	NA	NA
	S12	4.80 ± 0.45 ^Aa^	4.60 ± 0.55 ^Aa^	4.00 ± 0.00 ^Aa^	4.00 ± 0.71 ^ABa^	4.00 ± 0.00 ^Aa^	NA	NA	NA
	S21	3.20 ± 0.84 ^Ba^	3.00 ± 0.00 ^Ba^	3.00 ± 0.00 ^Ba^	2.80 ± 0.45 ^Ca^	3.00 ± 0.71 ^Ba^	NA	NA	NA
	S22	3.40 ± 0.55 ^Ba^	3.00 ± 0.00 ^Ba^	3.00 ± 0.00 ^Ba^	3.00 ± 0.71 ^BCa^	3.00 ± 0.71 ^Ba^	NA	NA	NA
	S31	6.80 ± 0.45 ^Ca^	6.60 ± 0.55 ^Ca^	6.00 ± 0.00 ^Cb^	6.00 ± 0.00 ^Db^	6.00 ± 0.00 ^Cb^	5.00 ± 0.00 ^Ac^	4.60 ± 0.55 ^Ac^	4.30 ± 0.45 ^Ac^
	S32	6.80 ± 0.45 ^Ca^	6.80 ± 0.55 ^Ca^	6.80 ± 0.45 ^Da^	6.20 ± 0.45 ^Da^	6.00 ± 0.00 ^Ca^	4.00 ± 0.00 ^Bb^	3.80 ± 0.84 ^Bb^	3.60 ± 0.55 ^Bb^
FISH ODOR	S11	1.40 ± 0.55 ^Aa^	1.60 ± 0.55 ^Aa^	2.00 ± 0.00 ^Ab^	2.00 ± 0.00 ^Ab^	2.00 ± 0.00 ^Ab^	NA	NA	NA
	S12	1.40 ± 0.55 ^Aa^	1.40 ± 0.55 ^Aa^	2.80 ± 0.45 ^Bb^	3.00 ± 0.00 ^Bb^	3.00 ± 0.00 ^Bb^	NA	NA	NA
	S21	1.20 ± 0.45 ^Aa^	1.20 ± 0.45 ^Aa^	1.20 ± 0.45 ^Ca^	1.40 ± 0.55 ^Ca^	2.00 ± 0.00 ^Ab^	NA	NA	NA
	S22	1.00 ± 0.00 ^Aa^	1.00 ± 0.00 ^Aa^	1.00 ± 0.00 ^Ca^	1.40 ± 0.55 ^Ca^	2.00 ± 0.00 ^Ab^	NA	NA	NA
	S31	0.20 ± 0.45 ^Ba^	0.60 ± 0.55 ^Aa^	0.80 ± 0.45 ^Ca^	0.80 ± 0.45 ^Da^	1.00 ± 0.00 ^Cab^	1.20 ± 0.45 ^Aab^	1.40 ± 0.55 ^Ab^	1.70 ± 0.45 ^Ab^
	S32	0.20 ± 0.45 ^Ba^	0.80 ± 0.45 ^Aa^	0.80 ± 0.45 ^Ca^	0.80 ± 0.45 ^Da^	1.00 ± 0.00 ^Ca^	2.00 ± 0.71 ^Bb^	2.38 ± 0.48 ^Bb^	2.40 ± 0.55 ^Bb^
RANCID ODOR	S11	0.00 ± 0.00 ^Aa^	0.00 ± 0.00 ^Aa^	0.00 ± 0.00 ^Aa^	0.40 ± 0.22 ^Aab^	0.60 ± 0.55 ^ABb^	NA	NA	NA
	S12	0.00 ± 0.00 ^Aa^	0.00 ± 0.00 ^Aa^	0.00 ± 0.00 ^Aa^	0.40 ± 0.22 ^Aab^	0.60 ± 0.55 ^ABb^	NA	NA	NA
	S21	0.00 ± 0.00 ^Aa^	0.00 ± 0.00 ^Aa^	0.00 ± 0.00 ^Aa^	0.60 ± 0.55 ^Ab^	0.80 ± 0.45 ^Ab^	NA	NA	NA
	S22	0.00 ± 0.00 ^Aa^	0.00 ± 0.00 ^Aa^	0.40 ± 0.22 ^Bab^	0.60 ± 0.55 ^Abc^	1.00 ± 0.00 ^Ac^	NA	NA	NA
	S31	0.00 ± 0.00 ^Aa^	0.00 ± 0.00 ^Aa^	0.00 ± 0.00 ^Aa^	0.00 ± 0.00 ^Aa^	0.00 ± 0.00 ^Ba^	0.60 ± 0.55 ^Aab^	0.80 ± 0.45 ^Ab^	0.80 ± 0.45 ^Ab^
	S32	0.00 ± 0.00 ^Aa^	0.00 ± 0.00 ^Aa^	0.00 ± 0.00 ^Aa^	0.00 ± 0.00 ^Aa^	0.00 ± 0.00 ^Ba^	0.60 ± 0.55 ^Aab^	0.80 ± 0.45 ^Ab^	0.80 ± 0.45 ^Ab^
FOREIGN ODOR	S11	0.00 ± 0.00 ^Aa^	0.00 ± 0.00 ^Aa^	0.00 ± 0.00 ^Aa^	0.00 ± 0.00 ^Aa^	0.20 ± 0.45 ^Aa^	NA	NA	NA
	S12	0.00 ± 0.00 ^Aa^	0.00 ± 0.00 ^Aa^	0.00 ± 0.00 ^Aa^	0.00 ± 0.00 ^Aa^	1.60 ± 0.55 ^Ba^	NA	NA	NA
	S21	0.00 ± 0.00 ^Aa^	2.00 ± 0.00 ^Bb^	2.00 ± 0.00 ^Bb^	2.00 ± 0.00 ^Bb^	2.40 ± 0.55 ^Cb^	NA	NA	NA
	S22	0.00 ± 0.00 ^Aa^	2.00 ± 0.00 ^Bb^	2.00 ± 0.00 ^Bb^	2.40 ± 0.55 ^Bb^	2.60 ± 0.55 ^Cb^	NA	NA	NA
	S31	0.00 ± 0.00 ^Aa^	0.00 ± 0.00 ^Aa^	0.00 ± 0.00 ^Aa^	0.00 ± 0.00 ^Aa^	0.00 ± 0.00 ^Aa^	0.10 ± 0.22 ^Aa^	0.10 ± 0.22 ^Aa^	0.10 ± 0.22 ^Aa^
	S32	0.00 ± 0.00 ^Aa^	0.00 ± 0.00 ^Aa^	0.00 ± 0.00 ^Aa^	0.00 ± 0.00 ^Aa^	0.00 ± 0.00 ^Aa^	0.60 ± 0.55 ^ABa^	0.80 ± 0.45 ^Ba^	0.80 ± 0.45 ^Ba^
CONSISTENCY	S11	3.00 ± 0.00 ^Aa^	3.00 ± 0.00 ^Aa^	3.00 ± 0.00 ^Aa^	3.60 ± 0.55 ^Aa^	4.20 ± 0.45 ^Ab^	NA	NA	NA
	S12	3.00 ± 0.00 ^Aa^	4.00 ± 0.00 ^Bb^	4.00 ± 0.00 ^Bb^	4.00 ± 0.00 ^ABb^	4.80 ± 0.45 ^Bb^	NA	NA	NA
	S21	4.00 ± 0.00 ^Ba^	4.00 ± 0.00 ^Ba^	4.60 ± 0.55 ^Ca^	4.20 ± 0.45 ^Ba^	4.80 ± 0.45 ^Ba^	NA	NA	NA
	S22	4.00 ± 0.00 ^Ba^	4.00 ± 0.00 ^Ba^	4.20 ± 0.45 ^Ba^	4.20 ± 0.45 ^Ba^	4.80 ± 0.45 ^Ba^	NA	NA	NA
	S31	3.20 ± 0.45 ^ABa^	3.60 ± 0.55 ^Cab^	4.00 ± 0.00 ^Bb^	4.00 ± 0.00 ^ABb^	4.00 ± 0.00 ^Ab^	4.20 ± 0.45 ^Ab^	4.20 ± 0.45 ^Ab^	4.20 ± 0.45 ^Ab^
	S32	3.60 ± 0.55 ^ABa^	3.60 ± 0.55 ^Ca^	3.80 ± 0.45 ^Ba^	4.00 ± 0.00 ^ABa^	4.00 ± 0.00 ^Aa^	4.00 ± 0.00 ^Aa^	4.00 ± 0.00 ^Aa^	4.00 ± 0.00 ^Aa^
ACIDITY	S11	2.60 ± 0.55 ^Aa^	3.00 ± 0.00 ^Aa^	4.00 ± 0.00 ^Ab^	4.00 ± 0.00 ^Ab^	4.20 ± 0.84 ^Ab^	NA	NA	NA
	S12	2.60 ± 0.55 ^Aa^	3.20 ± 0.45 ^Ab^	4.00 ± 0.00 ^Ac^	4.00 ± 0.00 ^Ac^	4.80 ± 0.45 ^Ac^	NA	NA	NA
	S21	3.40 ± 0.55 ^Ba^	4.00 ± 0.00 ^Bb^	4.00 ± 0.00 ^Ab^	4.20 ± 0.45 ^Ab^	4.60 ± 0.55 ^Ab^	NA	NA	NA
	S22	4.00 ± 0.00 ^Ba^	4.00 ± 0.00 ^Ba^	4.00 ± 0.00 ^Aa^	4.20 ± 0.45 ^Aa^	4.60 ± 0.55 ^Aa^	NA	NA	NA
	S31	5.00 ± 0.00 ^Ca^	5.00 ± 0.00 ^Ca^	5.00 ± 0.00 ^Ba^	5.00 ± 0.00 ^Ba^	5.00 ± 0.00 ^Ba^	5.00 ± 0.00 ^Aa^	5.20 ± 0.45 ^Aa^	5.20 ± 0.45 ^Aa^
	S32	5.00 ± 0.00 ^Ca^	5.00 ± 0.00 ^Ca^	5.00 ± 0.00 ^Ba^	5.00 ± 0.00 ^Ba^	5.00 ± 0.00 ^Ba^	5.00 ± 0.00 ^Aa^	5.80 ± 0.45 ^Bb^	5.75 ± 0.50 ^Bb^
SWEETNESS	S11	2.00 ± 0.00 ^Aa^	2.60 ± 0.55 ^Aab^	2.60 ± 0.55 ^Aab^	3.00 ± 0.00 ^Ab^	3.00 ± 0.71 ^Ab^	NA	NA	NA
	S12	2.40 ± 0.55 ^Aa^	2.60 ± 0.55 ^Aa^	2.60 ± 0.55 ^Aa^	3.00 ± 0.00 ^Aa^	3.00 ± 0.71 ^Aa^	NA	NA	NA
	S21	1.00 ± 0.00 ^Ba^	2.00 ± 0.00 ^Ab^	2.00 ± 0.00 ^Ab^	2.20 ± 0.45 ^Bb^	2.60 ± 0.55 ^Ab^	NA	NA	NA
	S22	1.00 ± 0.00 ^Ba^	2.00 ± 0.00 ^Ab^	2.00 ± 0.00 ^Ab^	2.00 ± 0.00 ^Bb^	2.60 ± 0.55 ^Ab^	NA	NA	NA
	S31	2.00 ± 0.00 ^Aa^	2.20 ± 0.45 ^Aa^	2.00 ± 0.00 ^Aa^	2.60 ± 0.55 ^ABab^	3.00 ± 0.00 ^Ab^	3.00 ± 0.00 ^Ab^	3.20 ± 0.45 ^Ab^	3.00 ± 0.00 ^Ab^
	S32	2.00 ± 0.00 ^Aa^	2.20 ± 0.45 ^Aa^	2.00 ± 0.00 ^Aa^	2.60 ± 0.55 ^ABab^	3.00 ± 0.00 ^Ab^	3.00 ± 0.00 ^Ab^	3.00 ± 0.00 ^Ab^	3.00 ± 0.00 ^Ab^
FISH AROMA	S11	1.40 ± 0.55 ^Aa^	1.60 ± 0.55 ^Aab^	2.20 ± 0.45 ^Ab^	3.00 ± 0.00 ^Ac^	3.00 ± 0.00 ^Ac^	NA	NA	NA
	S12	1.40 ± 0.55 ^Aa^	2.00 ± 0.00 ^Aa^	2.80 ± 0.45 ^Aa^	3.80 ± 0.45 ^Bb^	3.80 ± 0.45 ^Bb^	NA	NA	NA
	S21	1.00 ± 0.00 ^Aa^	1.00 ± 0.00 ^Ba^	2.00 ± 0.00 ^Ab^	2.80 ± 0.45 ^Ac^	3.20 ± 0.45 ^ABc^	NA	NA	NA
	S22	1.00 ± 0.00 ^Aa^	1.00 ± 0.00 ^Ba^	2.00 ± 0.00 ^Ab^	3.00 ± 0.71 ^Ac^	3.40 ± 0.55 ^ABc^	NA	NA	NA
	S31	1.00 ± 0.00 ^Aa^	1.00 ± 0.00 ^Ba^	1.00 ± 0.00 ^Ba^	1.00 ± 0.00 ^Ca^	1.00 ± 0.00 ^Ca^	1.00 ± 0.00 ^Aa^	1.20 ± 0.45 ^Aa^	1.40 ± 0.65 ^Aa^
	S32	1.00 ± 0.00 ^Aa^	1.00 ± 0.00 ^Ba^	1.00 ± 0.00 ^Ba^	1.00 ± 0.00 ^Ca^	1.00 ± 0.00 ^Ca^	1.20 ± 0.45 ^Aa^	1.50 ± 0.50 ^Aab^	2.00 ± 0.00 ^Ab^
CITRUS AROMA	S11	4.40 ± 0.55 ^Aa^	4.00 ± 0.00 ^Aa^	4.00 ± 0.00 ^Aa^	4.00 ± 0.00 ^Aa^	3.20 ± 0.45 ^Ab^	NA	NA	NA
	S12	4.40 ± 0.55 ^Aa^	4.00 ± 0.00 ^Aa^	4.00 ± 0.00 ^Aa^	4.00 ± 0.00 ^Aa^	3.40 ± 0.55 ^Ab^	NA	NA	NA
	S21	3.80 ± 0.45 ^Ba^	3.40 ± 0.55 ^Bab^	3.00 ± 0.00 ^Bb^	3.00 ± 0.00 ^Bb^	3.00 ± 0.00 ^ABb^	NA	NA	NA
	S22	3.60 ± 0.55 ^Ba^	3.60 ± 0.55 ^Ba^	3.00 ± 0.00 ^Bab^	3.00 ± 0.00 ^Bab^	2.60 ± 0.55 ^Bb^	NA	NA	NA
	S31	6.60 ± 0.55 ^Ca^	6.20 ± 0.45 ^Ca^	6.00 ± 0.00 ^Cab^	6.00 ± 0.00 ^Cab^	6.00 ± 0.00 ^Cab^	6.00 ± 0.71 ^Aab^	5.80 ± 0.45 ^Ab^	5.60 ± 0.55 ^Ab^
	S32	6.60 ± 0.55 ^Ca^	6.20 ± 0.45 ^Ca^	6.00 ± 0.00 ^Cab^	6.00 ± 0.00 ^Cab^	6.00 ± 0.00 ^Cab^	6.00 ± 0.71 ^Aab^	5.50 ± 0.87 ^Ab^	5.13±0.85 ^Ab^
RANCID TASTE	S11	0.00 ± 0.00 ^Aa^	0.00 ± 0.00 ^Aa^	0.00 ± 0.00 ^Aa^	0.00 ± 0.00 ^Aa^	0.60 ± 0.55 ^ABb^	NA	NA	NA
	S12	0.00 ± 0.00 ^Aa^	0.00 ± 0.00 ^Aa^	0.00 ± 0.00 ^Aa^	0.00 ± 0.00 ^Aa^	0.60 ± 0.55 ^ABb^	NA	NA	NA
	S21	0.00 ± 0.00 ^Aa^	0.00 ± 0.00 ^Aa^	0.00 ± 0.00 ^Aa^	0.60 ± 0.55 ^Bb^	1.00 ± 0.00 ^Ab^	NA	NA	NA
	S22	0.00 ± 0.00 ^Aa^	0.00 ± 0.00 ^Aa^	0.60 ± 0.55 ^Bb^	1.00 ± 0.00 ^Bb^	1.00 ± 0.00 ^Ab^	NA	NA	NA
	S31	0.00 ± 0.00 ^Aa^	0.00 ± 0.00 ^Aa^	0.00 ± 0.00 ^Aa^	0.00 ± 0.00 ^Aa^	0.00 ± 0.00 ^Ba^	0.00 ± 0.00 ^Aa^	0.40 ± 0.22 ^Ab^	0.60 ± 0.55 ^Ab^
	S32	0.00 ± 0.00 ^Aa^	0.00 ± 0.00 ^Aa^	0.00 ± 0.00 ^Aa^	0.00 ± 0.00 ^Aa^	0.00 ± 0.00 ^Ba^	0.00 ± 0.00 ^Aa^	0.40 ± 0.22 ^Ab^	0.60 ± 0.55 ^Ab^
AFTER TASTE	S11	0.80 ± 0.45 ^Aa^	1.00 ± 0.00 ^Aa^	1.20 ± 0.45 ^Aab^	1.40 ± 0.55 ^Ab^	1.40 ± 0.55 ^Ab^	NA	NA	NA
	S12	0.80 ± 0.45 ^Aa^	1.00 ± 0.00 ^Aa^	1.60 ± 0.55 ^Ab^	1.60 ± 0.55 ^Ab^	2.00 ± 0.00 ^Bb^	NA	NA	NA
	S21	1.80 ± 0.45 ^Ba^	1.80 ± 0.45 ^Ba^	2.20 ± 0.45 ^Bab^	2.40 ± 0.55 ^Bb^	3.60 ± 0.55 ^Cc^	NA	NA	NA
	S22	2.40 ± 0.55 ^Ca^	2.60 ± 0.55 ^Ca^	2.60 ± 0.55 ^BCa^	2.80 ± 0.45 ^BCa^	3.80 ± 0.45 ^Cb^	NA	NA	NA
	S31	1.80 ± 0.45 ^Ba^	2.60 ± 0.55 ^Cb^	2.60 ± 0.55 ^BCb^	3.20 ± 0.45 ^Cb^	3.60 ± 0.55 ^Cbc^	3.80 ± 0.45 ^Ac^	4.00 ± 0.00 ^Ac^	3.90 ± 0.55 ^Ac^
	S32	1.80 ± 0.45 ^Ba^	2.80 ± 0.45 ^Cb^	2.80 ± 0.45 ^Cb^	3.20 ± 0.45 ^Cbc^	3.60 ± 0.55 ^Cc^	4.00 ± 0.00 ^Ac^	4.00 ± 0.00 ^Ac^	4.13 ± 0.25 ^Ac^

*Note*: Different capital letters indicate significant differences between samples evaluated on the same day; small letters indicate differences on different days for the same sample (*p* < .05). S: Syrup form, C: Capsule form, G: Chewable form, NA: Not Applicable.

At the beginning of storage, the highest citrus odor was detected in S31‐S32 samples followed by S11‐S12 and S21‐S22, respectively. During storage, mostly there was no significant change until the end of day 29 (*p* > .05). Only for S31‐S32 samples on the last day of storage (day 49), there was a significant decrease in the citrus odor due to prolonged storage. Probably, due to the masking effect of citrus odor, the lowest fish odor was detected in samples S31‐S32 and the highest score was observed in S11‐S12 samples. In all samples, the fish odor scores increased during storage. On the other hand, rancid odor was not detected on the first days of storage for all samples, as expected, however, during the last days of storage very slight rancid odor was detected. The time of appearance was the latest in S31‐S32 samples which were detected to have a rancid odor starting from day 35. The earliest detection of rancid odor was in sample S22 which started to have a rancid odor on day 14.

Consistency of the samples slightly increased during storage being the highest for S21‐S22 for all time points. The highest acidity scores were found to be in S31‐S32 samples, and it did not change significantly during storage. However, for samples S11‐S12 and S21‐S22, there was a significant increase by the storage period. The perception of sweetness increased by the time but in general, the scores were not very high ranging between 1.0 and 3.2. On day 0, the fish aroma was detected at very low intensities between 1.0 and 1.4, but increased for S11‐S12 and S21‐S22 significantly during storage. However, there was no significant change for samples S31‐S32 throughout the whole storage period. On the contrary, the citrus aroma scores decreased for all samples by the time, but this decrease started at the latest in S31‐S32 samples on day 42. Rancid taste scores were quite similar to rancid odor scores, a slightly rancid taste was detected after certain time periods for each sample. The nature of aftertaste in all samples was different. In samples S11‐S12 and S31‐S32, the aftertaste was more like citrus aroma/flavor and slightly fishy, but for S21‐S22, the aftertaste was not pleasant and an irritating chemical‐like flavor was detected. Moreover, in samples S21‐S22, there was a chemical‐like taste that was described as undesirable and irritating by the panelists.

#### 3.4.2 Fish oil capsule

The sensory evaluation of fish oil capsules during storage period is given in Table 4. The fish oil capsules were investigated for their sensory attributes including fish odor, foreign odor, sweetness, and fish aroma. On the other hand, even though rancid odor and taste, soapy odor and taste, and vegetable oil taste were also evaluated by the panelists, these attributes were not detected at all throughout the whole panel sessions. Fish odor was detected at the highest levels in C21 and C22 samples which was observed to increase by the time and reached its highest value as 3 and 4 for C21 and C22, respectively. Similarly, foreign odor was detected as the highest in samples C21 and C22. The foreign odor was described as “fodder like” or “gelatine like” by the panelists. The lowest values were detected in C11 sample for all the time points. The foreign taste increased by the time for all samples which might be due to oxidation or the additive effect of increased fish odor during storage. On the last day of storage, there was no significant difference in the sweetness of samples for C11‐C12 and C21‐C22, but C31‐C32 was significantly higher than these samples. Fish aroma of the samples increased by the time, and at the end of storage, the highest score was observed in C22 sample. On the other hand, it was observed that the samples belonging to the same company but provided by different stores may indicate significant differences in some of the sensory attributes which is an indicator of nonstandard raw material, ingredient, or processing. Capsulated samples were better protected according to the sensory evaluation scores since no rancid odor and flavor was detected in samples indicating that the encapsulating material provided a good protection against oxidation.

**TABLE 4:** Sensory evaluation of fish oil capsules

TERMS	SAMPLE	DAYS				
		0	7	14	21	29
FISH ODOR	C11	1.20 ± 0.45 ^Aa^	1.60 ± 0.55 ^Aa^	1.60 ± 0.55 ^Aa^	1.60 ± 0.55 ^Aa^	1.60 ± 0.55 ^Aa^
	C12	1.80 ± 0.45 ^ABa^	2.00 ± 0.71 ^ABa^	2.00 ± 0.00 ^ABa^	2.20 ± 0.45 ^ABab^	3.00 ± 0.10 ^Bb^
	C21	2.00 ± 0.71 ^ABa^	2.40 ± 0.55 ^ABab^	2.00 ± 0.00 ^ABa^	2.60 ± 0.55 ^ABab^	3.00 ± 0.10 ^Bb^
	C22	2.60 ± 0.55 ^Ba^	3.20 ± 0.84 ^Bab^	3.00 ± 0.00 ^Cab^	3.00 ± 0.71 ^Bab^	4.00 ± 0.00 ^Cb^
	C31	2.00 ± 0.00 ^ABa^	2.20 ± 0.45 ^ABab^	2.60 ± 0.55 ^BCb^	2.60 ± 0.55 ^ABb^	2.40 ± 0.55 ^Bb^
	C32	2.00 ± 0.00 ^ABa^	2.40 ± 0.55 ^ABa^	2.20 ± 0.45 ^ABa^	2.20 ± 0.45 ^ABa^	2.40 ± 0.55 ^ABa^
FOREIGN ODOR	C11	0.00 ± 0.00 ^Aa^	0.80 ± 0.45 ^Ab^	1.00 ± 0.00 ^Ab^	1.00 ± 0.00 ^Ab^	2.00 ± 0.00 ^Ac^
	C12	1.00 ± 0.00 ^Ba^	1.00 ± 0.00 ^Aa^	1.40 ± 0.89 ^Aab^	2.00 ± 0.00 ^Bb^	3.20 ± 0.45 ^Ab^
	C21	3.00 ± 0.71 ^Ca^	3.20 ± 0.45 ^Ba^	3.60 ± 0.55 ^BCa^	3.80 ± 0.84^CDa^	4.00 ± 0.20 ^Ba^
	C22	3.60 ± 0.55 ^Ca^	4.00 ± 0.00 ^Cab^	4.20 ± 0.45 ^Bb^	4.20 ± 0.45 ^Cc^	5.00 ± 0.00 ^Cd^
	C31	1.80 ± 0.45 ^Ba^	2.80 ± 0.45 ^Bb^	3.00 ± 0.00 ^Cb^	3.00 ± 0.00 ^Db^	3.00 ± 0.20 ^Db^
	C32	1.80 ± 0.45 ^Ba^	3.00 ± 0.00 ^Bb^	3.00 ± 0.00 ^Cb^	3.20 ± 0.45 ^Db^	3.40 ± 0.55 ^Db^
SWEETNESS	C11	2.80 ± 0.45 ^Aa^	2.40 ± 0.89 ^Aa^	2.00 ± 0.00 ^Ab^	1.80 ± 0.45 ^Ab^	2.00 ± 0.15 ^Ab^
	C12	2.80 ± 0.45 ^Aa^	2.00 ± 0.71 ^ABbc^	1.80 ± 0.45 ^Ac^	2.00 ± 0.00 ^ABb^	2.00 ± 0.15 ^Ab^
	C21	1.40 ± 0.55 ^Ba^	1.80 ± 0.45 ^Bab^	2.00 ± 0.00 ^Ab^	2.20 ± 0.45 ^ABb^	2.00 ± 0.00 ^Ab^
	C22	1.40 ± 0.55 ^Ba^	1.80 ± 0.45 ^Bab^	2.00 ± 0.71 ^Ab^	2.40 ± 0.55 ^Bc^	2.00 ± 0.15 ^Ab^
	C31	2.20 ± 0.45 ^Ca^	2.20 ± 0.45 ^Aa^	3.00 ± 0.00 ^Bb^	3.20 ± 0.45 ^Cb^	3.00 ± 0.00 ^Bb^
	C32	2.20 ± 0.45 ^Ca^	2.40 ± 0.55 ^Aa^	3.00 ± 0.00 ^Bb^	3.20 ± 0.45 ^Cb^	3.00 ± 0.15 ^Bb^
FISH AROMA	C11	1.00 ± 0.00 ^ABab^	0.60 ± 0.55 ^Aa^	1.00 ± 0.00 ^Aab^	1.20 ± 0.45 ^Ab^	1.20 ± 0.20 ^Aab^
	C12	0.80 ± 0.45 ^Aa^	1.20 ± 0.45 ^Bb^	2.00 ± 0.00 ^Bc^	2.00 ± 0.71 ^Bc^	3.00 ± 0.00 ^Bd^
	C21	2.60 ± 0.55 ^Ca^	2.80 ± 0.45 ^Ca^	3.00 ± 0.00 ^Cab^	3.40 ± 0.89 ^Cb^	3.00 ± 0.00 ^Bab^
	C22	2.80 ± 0.45 ^Ca^	3.20 ± 0.45 ^Dab^	3.40 ± 0.55 ^Cb^	3.20 ± 0.45 ^Cab^	4.00 ± 0.00 ^Cc^
	C31	1.40 ± 0.89 ^Ba^	1.60 ± 0.55 ^Bab^	1.60 ± 0.89 ^ABab^	2.00 ± 0.00 ^Bb^	2.40 ± 0.89 ^Bc^
	C32	1.60 ± 0.55 ^Ba^	1.60 ± 0.55 ^Ba^	1.60 ± 0.89 ^ABa^	2.00 ± 0.00 ^Bb^	2.40 ± 0.89 ^Bc^

*Note*: Different capital letters indicate significant differences between samples evaluated on the same day; small letters indicate differences on different days for the same sample (*p* < .05). S: Syrup form, C: Capsule form, G: Chewable form.

#### 3.4.3 Fish oil chewable form

The panelists were asked to evaluate several sensory attributes in gel samples but rancid odor and taste, soapy odor and taste were not detected at all throughout the whole panel, and thus removed from the list. The rest of the evaluated attributes are listed in Table 5. According to the results, the fruity odor either did not change significantly during storage or decreased in different samples. The decrease in the fruity odor scores can be explained by the increase in other odors including fish odor or foreign odor which could have suppressed the fruity odor. The highest scores for fruity odor were obtained for G11‐G12 samples which was significantly higher than the other samples. On the other hand, these samples (G11‐G12) were detected to have the lowest fish odor within all samples. It is highly probable that the high fruity odor masked the fish odor efficiently. Except for G11 sample, rest of the samples showed a significant increase in their fish odor at the end of storage compared to Day 0. G11 and G12 samples had no foreign odor, and G31‐G32 samples had only a slightly foreign odor, but G21‐G22 samples were indicated to have high scores for foreign odor which significantly increased during storage. The panelists described this foreign odor as “herbal.” With respect to chewiness, G11‐G12 samples were not chewy at all according to the panelists. On the other hand, for G21‐G22 samples, the chewiness increased by the time and reached its maximum value on day 29. The chewiness for G21‐G22 was the highest, and the difference in this attribute as compared with G31‐G32 was significant (*p* < .05). The hardness of the samples did not change significantly during storage. The highest sweet taste was observed in G21‐G22 samples followed by G11‐G12 samples and there was no significant difference between these samples. G31‐G32 samples had the lowest sweetness scores and these values did not change significantly during storage at any time point. Compared to fish odor, fish aroma showed a different trend. Even though G11‐G12 samples had the lowest fish odor scores, they showed the highest fish aroma attribute followed by G21‐G22, and G31‐G32. The fish aroma of samples mostly did not change significantly during storage. Except for G11 sample, the fruity aroma of all samples decreased significantly at the end of their storage period. The fruity aroma of samples G11‐G12 was the highest among all similar to that of fruity odor. The aftertaste of all samples was significantly increased by the time; however, their characteristics were different from each other. In G11‐G12 samples, panelists detected a fruity aftertaste. On the other hand, in G21‐G22, the aftertaste was described as herbal, and in G31‐G32 samples, it was described as a sharp/strong fruity aroma.

**TABLE 5:** Sensory evaluation of fish oil in chewable forms

TERMS	SAMPLE	DAYS				
		0	7	14	21	29
FRUITY ODOR	G11	4.40 ± 0.55 ^Aa^	4.40 ± 0.89 ^Aa^	4.40 ± 0.55 ^Aa^	3.80 ± 0.45 ^Ab^	3.40 ± 0.55 ^Ab^
	G12	4.40 ± 0.55 ^Aa^	4.60 ± 0.89 ^Aa^	4.60 ± 0.55 ^Aa^	3.75 ± 0.50 ^Ab^	3.40 ± 0.55 ^Ab^
	G21	1.80 ± 0.45 ^Ba^	1.20 ± 0.45 ^BAb^	1.00 ± 0.00 ^Bb^	1.20 ± 0.45 ^BAb^	1.00 ± 0.00 ^Bb^
	G22	1.60 ± 0.00 ^Ba^	1.20 ± 0.45 ^BAb^	1.00 ± 0.00 ^Bb^	1.00 ± 0.00 ^Bb^	1.00 ± 0.00 ^Bb^
	G31	2.00 ± 0.00 ^Ba^	2.00 ± 0.00 ^Ca^	2.00 ± 0.00 ^Ca^	2.20 ± 0.45 ^Ca^	2.00 ± 0.00 ^Ca^
	G32	2.00 ± 0.00 ^Ba^	2.00 ± 0.00 ^Ca^	2.00 ± 0.00 ^Ca^	2.20 ± 0.45 ^Ca^	2.00 ± 0.00 ^Ca^
FISH ODOR	G11	0.00 ± 0.00 ^Aa^	0.00 ± 0.00 ^Aa^	0.00 ± 0.00 ^Aa^	0.20 ± 0.45 ^Aa^	0.20 ± 0.45 ^Aa^
	G12	0.00 ± 0.00 ^Aa^	0.00 ± 0.00 ^Aa^	0.00 ± 0.00 ^Aa^	0.20 ± 0.45 ^Aa^	0.40 ± 0.89 ^Ab^
	G21	2.20 ± 0.45 ^Ba^	2.40 ± 0.45 ^BAb^	2.40 ± 0.45 ^BAb^	2.40 ± 0.55 ^BAb^	2.60 ± 0.55 ^Bb^
	G22	2.40 ± 0.55 ^Ba^	2.40 ± 0.89 ^Ba^	2.60 ± 0.55 ^BAb^	2.60 ± 0.55 ^BAb^	2.80 ± 0.45 ^Bcb^
	G31	2.20 ± 0.45 ^Ba^	2.20 ± 0.84 ^Ba^	2.80 ± 0.45 ^BAb^	2.80 ± 0.45 ^BCAb^	30 ± 0.00 ^Cb^
	G32	2.20 ± 0.45 ^Ba^	2.40 ± 0.89 ^Ba^	2.80 ± 0.45 ^BAb^	3.00 ± 0.00 ^Cb^	3.00 ± 0.00 ^Cb^
FOREIGN ODOR	G11	0.00 ± 0.00 ^Aa^	0.00 ± 0.00 ^Aa^	0.00 ± 0.00 ^Aa^	0.00 ± 0.00 ^Aa^	0.00 ± 0.00 ^Aa^
	G12	0.00 ± 0.00 ^Aa^	0.00 ± 0.00 ^Aa^	0.00 ± 0.00 ^Aa^	0.00 ± 0.00 ^Aa^	0.00 ± 0.00 ^Aa^
	G21	3.60 ± 0.55 ^Ba^	4.40 ± 0.55 ^Bb^	4.80 ± 0.45 ^BBc^	5.00 ± 0.00 ^Bc^	5.00 ± 0.00 ^Bc^
	G22	2.80 ± 0.84 ^Ca^	4.00 ± 0.00 ^Bb^	4.00 ± 0.00 ^Cb^	4.40 ± 0.55 ^CBc^	4.60 ± 0.55 ^Bc^
	G31	0.00 ± 0.00 ^Aa^	0.20 ± 0.45 ^ACa^	0.80 ± 0.45 ^Db^	1.00 ± 0.00 ^Db^	1.00 ± 0.00 ^Cb^
	G32	0.00 ± 0.00 ^Aa^	0.60 ± 0.55 ^Cb^	0.80 ± 0.45 ^Db^	1.00 ± 0.00 ^DBc^	1.00 ± 0.00 ^CBc^
CHEWINESS	G11	0.00 ± 0.00 ^Aa^	0.00 ± 0.00 ^Aa^	0.00 ± 0.00 ^Aa^	0.00 ± 0.00 ^Aa^	0.00 ± 0.00 ^Aa^
	G12	0.00 ± 0.00 ^Aa^	0.00 ± 0.00 ^Aa^	0.00 ± 0.00 ^Aa^	0.00 ± 0.00 ^Aa^	0.00 ± 0.00 ^Aa^
	G21	5.00 ± 0.00 ^Ba^	5.20 ± 0.45 ^Ba^	5.60 ± 0.55 ^BAb^	6.00 ± 0.00 ^Bb^	6.00 ± 0.00 ^Bb^
	G22	5.20 ± 0.45 ^Ba^	5.20 ± 0.45 ^Ba^	5.60 ± 0.55 ^BAb^	6.00 ± 0.00 ^Bb^	6.00 ± 0.00 ^Bb^
	G31	2.00 ± 0.00 ^Ca^	2.00 ± 0.00 ^Ca^	2.00 ± 0.00 ^Ca^	2.20 ± 0.45 ^Ca^	2.00 ± 0.00 ^Ca^
	G32	2.00 ± 0.00 ^Ca^	2.00 ± 0.00 ^Ca^	2.00 ± 0.00 ^Ca^	2.20 ± 0.45 ^Ca^	2.00 ± 0.00 ^Ca^
SWEETNESS	G11	5.20 ± 0.45 ^Aa^	5.20 ± 0.84 ^Aa^	5.20 ± 0.45 ^Aa^	5.20 ± 0.45 ^Aa^	4.60 ± 0.55 ^Ab^
	G12	5.20 ± 0.45 ^Aa^	5.20 ± 0.84 ^Aa^	5.20 ± 0.45 ^Aa^	5.25 ± 0.50 ^Aa^	4.60 ± 0.55 ^Ab^
	G21	5.80 ± 0.45 ^Ba^	5.80 ± 0.45 ^Ba^	5.40 ± 0.55 ^BAb^	5.40 ± 0.55 ^AAb^	5.20 ± 0.45 ^Bb^
	G22	5.80 ± 0.45 ^Ba^	5.60 ± 0.55 ^ABAb^	5.40 ± 0.55 ^BAb^	5.60 ± 0.55 ^AAb^	5.20 ± 0.45 ^Bb^
	G31	3.40 ± 0.55 ^Ca^	3.60 ± 0.55 ^Ca^	3.80 ± 0.45 ^Ca^	3.80 ± 0.45 ^Ca^	3.80 ± 0.45 ^Ca^
	G32	3.40 ± 0.55 ^Ca^	3.60 ± 0.55 ^Ca^	3.80 ± 0.45 ^Ca^	3.80 ± 0.45 ^Ca^	3.80 ± 0.45 ^Ca^
FISH AROMA	G11	4.40 ± 0.55 ^Aa^	4.60 ± 0.55 ^Aa^	4.80 ± 0.45 ^Aa^	4.80 ± 0.45 ^Aa^	4.80 ± 0.45 ^Aa^
	G12	4.60 ± 0.55 ^Aa^	4.60 ± 0.55 ^Aa^	4.75 ± 0.50 ^Aa^	5.00 ± 0.00 ^Aa^	5.00 ± 0.71 ^Aa^
	G21	2.20 ± 0.84 ^BCa^	2.60 ± 0.89 ^BCa^	3.00 ± 0.00 ^BAb^	3.20 ± 0.45 ^Bb^	3.60 ± 0.55 ^Bb^
	G22	2.60 ± 0.89 ^Ba^	2.80 ± 0.45 ^Ba^	3.00 ± 0.71 ^Ba^	3.80 ± 0.45 ^Cb^	3.80 ± 0.45 ^Bb^
	G31	1.80 ± 0.45 ^Ca^	2.00 ± 0.00 ^Ca^	2.20 ± 0.45 ^Ca^	2.20 ± 0.84 ^Da^	2.20 ± 0.45 ^Ca^
	G32	1.80 ± 0.45 ^Ca^	2.00 ± 0.00 ^Ca^	2.20 ± 0.45 ^Ca^	2.20 ± 0.84 ^Da^	2.20 ± 0.45 ^Ca^
FRUITY AROMA	G11	3.40 ± 0.55 ^Aa^	3.00 ± 0.71 ^Aa^	3.20 ± 0.45 ^Aa^	3.00 ± 0.00 ^Aa^	3.00 ± 0.71 ^Aa^
	G12	3.80 ± 0.45 ^ABa^	3.80 ± 0.45 ^Ba^	3.20 ± 0.84 ^AAb^	3.25 ± 0.50 ^Bb^	3.20 ± 0.45 ^Ab^
	G21	4.80 ± 0.55 ^Ca^	4.20 ± 0.84 ^Bcb^	2.80 ± 0.45 ^Ac^	2.60 ± 0.89 ^Ac^	2.60 ± 0.55 ^Bc^
	G22	4.80 ± 0.55 ^Ca^	4.40 ± 0.89 ^Cb^	2.80 ± 0.45 ^Ac^	2.60 ± 0.89 ^Ac^	2.60 ± 0.55 ^Bc^
	G31	4.00 ± 0.71 ^Ba^	3.80 ± 0.45 ^Ba^	3.20 ± 0.45 ^Ab^	3.00 ± 0.00 ^Ab^	2.20 ± 0.45 ^Bc^
	G32	4.00 ± 0.71 ^Ba^	3.80 ± 0.45 ^Ba^	3.20 ± 0.45 ^Ab^	3.00 ± 0.00 ^Ab^	2.40 ± 0.55 ^Bc^
AFTER TASTE	G11	1.80 ± 0.45 ^Aa^	2.00 ± 0.00 ^Aa^	2.00 ± 0.00 ^Aa^	2.80 ± 0.45 ^Ab^	3.00 ± 0.71 ^Ab^
	G12	2.00 ± 0.00 ^ABa^	2.20 ± 0.84 ^ABa^	2.40 ± 0.55 ^ABAb^	2.60 ± 0.55 ^Ab^	3.20 ± 0.45 ^Ac^
	G21	2.40 ± 0.55 ^Ba^	2.60 ± 0.55 ^Ba^	2.80 ± 0.45 ^BAb^	3.00 ± 0.00 ^Ab^	3.40 ± 0.55 ^ABb^
	G22	2.40 ± 0.55 ^Ba^	2.80 ± 0.45 ^BAb^	3.00 ± 0.00 ^Bb^	3.00 ± 0.00 ^Ab^	3.60 ± 0.55 ^Bc^
	G31	2.00 ± 0.00 ^ABa^	2.20 ± 0.45 ^ABa^	2.40 ± 0.55 ^ABAb^	2.80 ± 0.45 ^Ab^	2.80 ± 0.84 ^BAb^
	G32	2.00 ± 0.00 ^ABa^	2.40 ± 0.55 ^ABAb^	2.80 ± 0.45 ^Bb^	2.80 ± 0.84 ^Ab^	3.40 ± 0.55 ^ABc^

*Note*: Different capital letters indicate significant differences between samples evaluated on the same day; small letters indicate differences on different days for the same sample (*p* < .05). S: Syrup form, C: Capsule form, G: Chewable form.

In short, samples were mostly different from each other in terms of their taste, aroma, and texture. Specifically, the differences between the aromas of samples made the comparison difficult, some of which had an herbal aroma, whereas the others had fruity aroma. On the other hand, different textural characteristics may also lead to differences in the evaluation of other attributes.

## 4 CONCLUSION

Different forms of omega‐3 products (3 capsules, 3 chewable forms, and 3 syrups) offered for sale in the Turkish market with the highest sales figures were selected and the products were stored in their own packages at room temperature under dark conditions. Comparative oxidation analysis and sensory analysis were performed once a week, including the properties at the time of first opening. GOED provides limit values for PV as 5 meq/kg oil, for *p*‐AV as 20, and for TOTOX as 26. For PV, at day 0, only G1 and G2 samples were out of the limit values among all the products (G11: 6.25, G12: 12.22, G21: 6.76, and G22: 6.35 meq/kg oil). On the other hand, only G3 samples were not over the PV limit values at the end of the storage period. When *p*‐AV values of the samples were compared, there were no samples having a higher value than the limit value at neither the beginning nor the end of the storage period. However, sample S21 showed a nonlinear trend and did not comply with the limits on days 7 and 14, with *p*‐AV values of 21.62 and 23.48, respectively. For TOTOX values, all samples were complied with the limit values at the first opening while 56.3% of the samples were out of limits at the end of the storage period.

Variations in some of the sensory attributes were found in the analysis of omega‐3 products with different production dates and lot numbers, although they are the products of the same company.

Capsulated samples were better protected according to the sensory evaluation scores since no rancid odor and flavor were detected in samples indicating that the encapsulating material provided a good protection against oxidation. While an increase in oxidation parameters was observed in different forms of fish oils with storage, it was observed that individual packaging had a protective effect against oxidation.

## CONFLICT OF INTEREST

The authors declare no conflict of interest.

## Data Availability

The data that support the findings of this study are available on request from the corresponding author.
